# Periodontitis from Pressure Anesthesia

**Published:** 1917-08

**Authors:** 


### PERIODONTITIS FROM PRESSURE ANESTHESIA.

In the days when I had so much trouble I was using a pellet of cocaine and adrenalin. This was very effective so far as anesthesia was concerned, and there was no very troublesome hemorrhage at that sitting. But I frequently had tender teeth. Finally I concluded that the adrenalin was the cause. By constricting the capillaries the bothersome bleeding was controlled, but a few hours later, when its effect would wear off, what occurred? The congested capillaries emptied themselves, and as a result we had an extravasation of blood about the apex, with pressure and pain, which would continue until this blood clot were accounted for in some manner. Likewise this free blood afforded an excellent medium for germs, and, consequently, actually invited bacterial invasion. I abandoned adrenalin, mixed my cocaine with sterile water, and my apical troubles ceased.

I presume that ninety per cent. of dentists after pulp removal dress the canal with cotton charged with some medicine, usually one of the essential oils. Now, when the secondary hemorrhage occurs, it can not enter the canal of the tooth, because that is already choked up with cotton loaded with an oil. Hence the blood clot forms at the end of the root, with pressure and discomfort, if not actual pain. We have a pericementitis which may develop into an acute abscess if not promptly and effectively treated.

After removing the pulp, or the major part of the pulp, as the case may be, make no effort to control the hemorrhage. On the contrary, encourage the bleeding, because the more the capillaries are depleted the less likelihood will there be of a secondary hemorrhage.

If the bleeding is very profuse, I always suspect that there is a stump of the pulp still remaining. Indeed, as no one can look at an extracted pulp and determine whether it has been removed entire or not, it is safest always to proceed as though some were still remaining. It will be much easier to remove it before the anesthesia subsides than it will be a day later. Therefore I use a special broach, which has but two tiny barbs at the extreme tip, and with this I search for any remainder of the pulp that may still be present. Often I bring away with this instrument a millimeter or more of pulp tissue. Again the hemorrhage is encouraged rather than checked, and when all bleeding has ceased, the tooth is dressed and temporarily sealed up. —Dr. Ottolengui in *Dental Items of Interest.*

